# Correction: Fast and scalable solvent-free access to Lappert's heavier tetrylenes E{N(SiMe_3_)_2_}_2_ (E = Ge, Sn, Pb) and ECl{N(SiMe_3_)_2_} (E = Ge, Sn)

**DOI:** 10.1039/d3sc90211k

**Published:** 2023-11-03

**Authors:** Javier A. Cabeza, Javier F. Reynes, Felipe García, Pablo García-Álvarez, Rubén García-Soriano

**Affiliations:** a Departamento de Química Orgánica e Inorgánica-IUQOEM, Centro de Innovación en Química Avanzada (ORFEO-CINQA), Universidad de Oviedo 33071 Oviedo Spain garciafelipe@uniovi.es pga@uniovi.es; b School of Chemistry, Monash University Clayton Victoria 3800 Australia Felipe.Garcia@monash.edu

## Abstract

Correction for ‘Fast and scalable solvent-free access to Lappert's heavier tetrylenes E{N(SiMe_3_)_2_}_2_ (E = Ge, Sn, Pb) and ECl{N(SiMe_3_)_2_} (E = Ge, Sn)’ by Javier A. Cabeza *et al.*, *Chem. Sci.*, 2023, https://doi.org/10.1039/D3SC02709K.

The originally published Fig. 1 and 2 omitted details relevant to the shown synthesis.

**Fig. 1:**
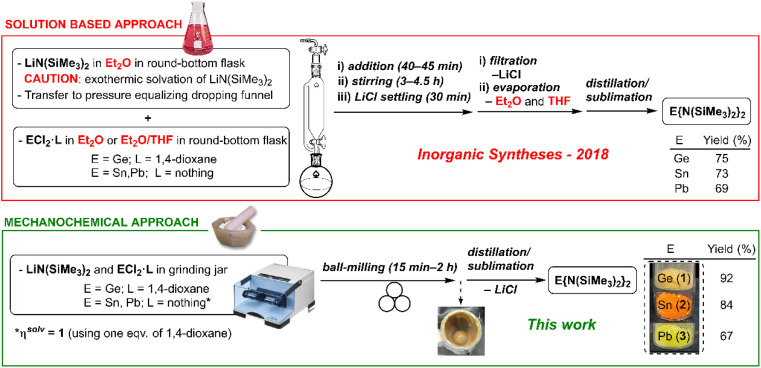
Conventional solution-based (ref. 24*c*) *vs.* mechanochemical syntheses (this work) for compounds **1–3**.

**Fig. 2:**
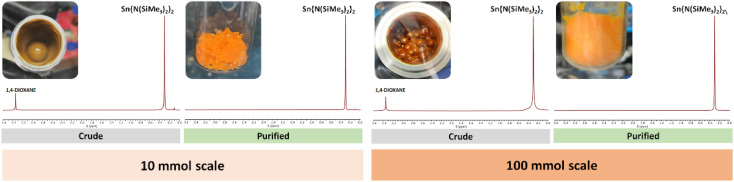
Side-by-side comparison of the 10 and 100 mmol scale reactions (left and right, respectively) for the synthesis of Sn{N(SiMe_3_)_2_}_2_ (**2**). The inserts display the crude product obtained in 10 mL and the 250 mL stainless steel jars, and the resulting isolated product stored in a 20 mL vial and a 100 mL Schlenk tube for the small and large scales, respectively. See ESI† for expanded NMRs.

In Fig. 1, the further details associated with the asterisk, *η^solv^ = 1 (using one eq. of 1,4-dioxane), was omitted.

In Fig. 2, the inserted NMR spectra were incorrect.

The updated Fig. 1 and 2 within this Correction show the intended graphics and replace those in the original manuscript.

The Royal Society of Chemistry apologises for these errors and any consequent inconvenience to authors and readers.

## Supplementary Material

